# Bodily motion fluctuation improves reaching success rate in a neurophysical agent via geometric-stochastic resonance

**DOI:** 10.1371/journal.pone.0188298

**Published:** 2017-12-08

**Authors:** Shogo Yonekura, Yasuo Kuniyoshi

**Affiliations:** The University of Tokyo, Tokyo, Japan; Southwest University, CHINA

## Abstract

Organisms generate a variety of noise types, including neural noise, sensory noise, and noise resulting from fluctuations associated with movement. Sensory and neural noises are known to induce stochastic resonance (SR), which improves information transfer to the subjects control systems, including the brain. As a consequence, sensory and neural noise provide behavioral benefits, such as stabilization of posture and enhancement of feeding efficiency. In contrast, the benefits of fluctuations in the movements of a biological system remain largely unclear. Here, we describe a novel type of noise-induced order (NIO) that is realized by actively exploiting the motion fluctuations of an embodied system. In particular, we describe the theoretical analysis of a feedback-controlled embodied agent system that has a geometric end-effector. Furthermore, through several numerical simulations we demonstrate that the ratio of successful reaches to goal positions and capture of moving targets are improved by the exploitation of motion fluctuations. We report that reaching success rate improvement (RSRI) is based on the interaction of the geometric size of an end-effector, the agents motion fluctuations, and the desired motion frequency. Therefore, RSRI is a geometrically induced SR-like phenomenon. We also report an interesting result obtained through numerical simulations indicating that the agents neural and motion noise must be optimized to match the prey’s motion noise in order to maximize the capture rate. Our study provides a new understanding of body motion fluctuations, as they were found to be the active noise sources for a behavioral NIO.

## Introduction

The bodily movements of a biological system are noisy because of the stochastic nature of biological sensory, neural, and actuation systems. Sensory and neural noise induce stochastic resonance (SR), which provides a variety of benefits to an organism. These benefits include improvements in information transmission to and through the neural system. Furthermore, sensory and neural noise enhance cognitive performance [1, 2], reflexes [3], feeding [4–6], stochastic action selection [7], and memory-perception balance [8, 9] (for other benefits of SR, see the following reviews [10–12]). In contrast to the tremendous known benefits of neural and sensory noise, the benefits of the noise inherent in motion fluctuations are largely unclear. In fact, only a few SR-like benefits have been proposed for this type of noise (e.g., improvements in visual acuity due to eye tremor [13]).

The major reason that motion fluctuation is not considered to be a source of behavioral SR or other noise-induced order (NIO) is that it exhibits a Lorentz-type spectrum and long-term correlation. In fact, it has been shown that colored noise in general degrades the SR effect in a nonlinear system [14, 15]. Because the motion fluctuations of relatively large-bodied animals, such as mammals, reptiles, and fish mostly have long correlation times, they cannot be used directly as sources of noise to induce SR in a nonlinear system. However, these motion fluctuations can still be helpful for a neurophysical agent. We find that by using the measure of reaching success rate, we can observe a novel kind of NIO. Furthermore, we report that the bodily motion fluctuations of a neurophysical agent provide aperiodic and stochastic input signals to a feedback motion controller consisting of neurons. This leads to the emergence of neural aperiodic SR [16–18].

In the following article, we report the results of our theoretical analysis of the reaching success rate improvement (RSRI) of a Brownian particle that is controlled to reach a periodically-moving target. Next, we demonstrate that the RSRI ratio is dependent on the geometric size of an end-effector used to catch a target. We further show the RSRI is a novel NIO based on the mechanism of geometric-stochastic resonance (GSR), wherein the geometric size of the end-effector, the frequency of the target movement, and the motion noise intensities interact with each other and improve reaching success ratio. As an applicative and more general experimental framework of GSR, we consider a numerically-simulated neurophysical agent with a two-dimensional body and a neural motion controller consisting of two arrays of FitzHugh-Nagumo neurons. Furthermore, we consider two experimental setups implemented using numerical simulations: a static reaching task, wherein the agent tracks along a predesigned path, and a dynamic capturing task, wherein the agent captures randomly moving objects.

## Methods

### Theoretical basis of RSRI via GSR

**Fig 1:**
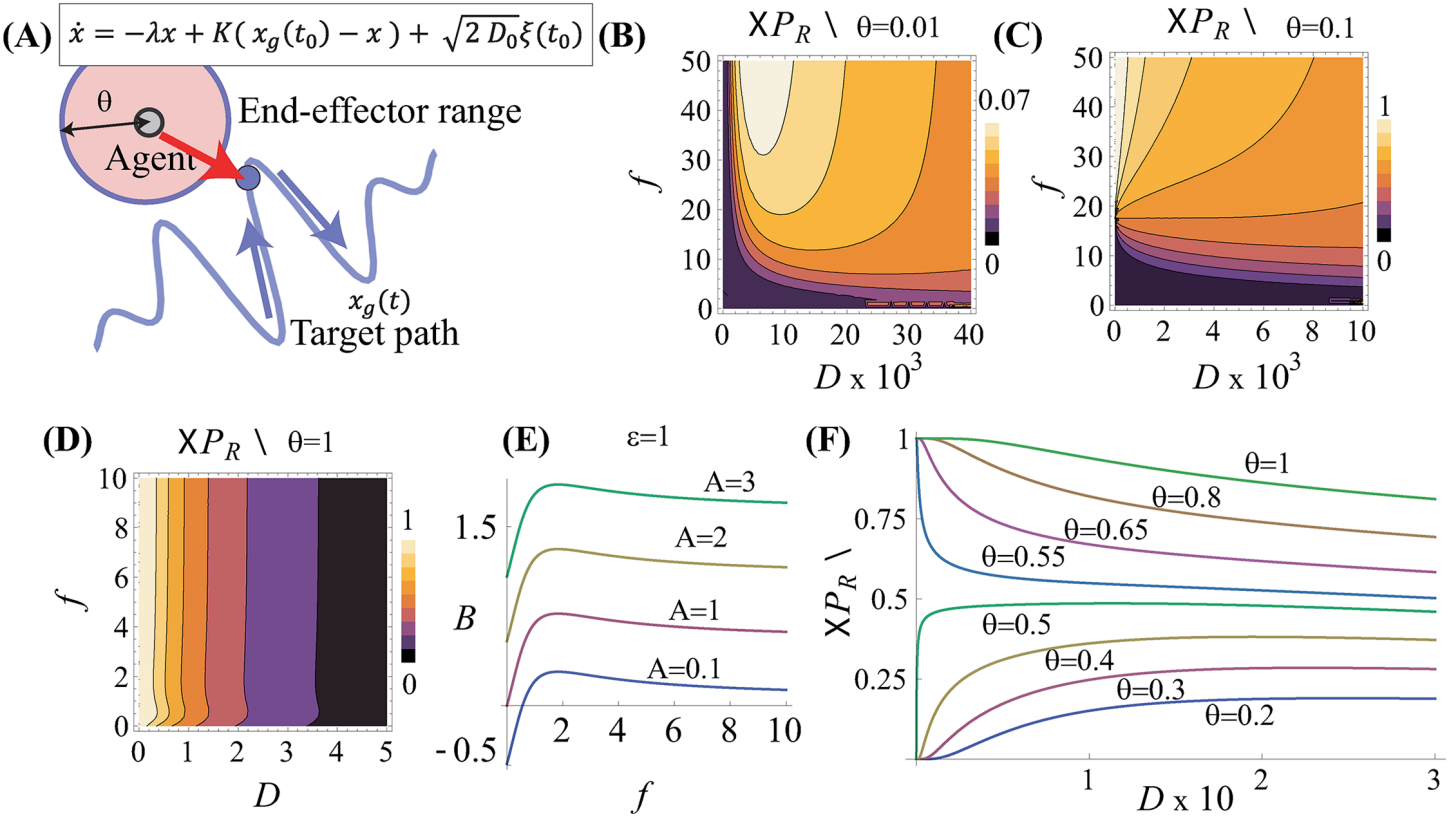
Theoretical analysis of RSRI. **(A)** Schematic model of a feedback-controlled Brownian particle agent. The agent has an end-effector of size *θ* used to reach a target moving along the pre-designed path *x*_*g*_(*t*). For simplicity, we assume that *x*_*g*_(*t*) is periodic. **(B,C,D)** Plot of theoretical 〈*P*_*R*_〉 with contour lines versus the moving target frequency *f* and the agent motion noise intensity *D* computed using Eq (5) with *θ* = 0.01 **(B)**, *θ* = 0.1 **(C)**, and *θ* = 1 **(D)**. **(E)** *B* with respect to *f* and *A* = 0.1, 1, 2, 3 with *ϵ* = 1. Note that with *t* = 1/*f*, lim_*f*→∞_ *B* = *A* cos(1). **(F)** 〈*P*_*R*_〉 with respect to *D* × 10 and *θ* = 0.2, 0.4, 0.5, 0.55, 0.65, 0.8, 1 with *A* = 0.1.

We consider an overdamped Brownian particle driven by a feedback controller as

$$\begin{array}{c} \dot{x}=-\lambda x+K\left(x_{g}\left(t_{0}\right)-x\right)+\sqrt{2D_{0}}\xi \left(t_{0}\right), \end{array}$$ (1)

where *x* is the position of the particle, *x*_*g*_ is the pre-designed goal position at time *t*_0_ and *x*_*g*_(*t*_0_) = *ϵ*_0_ cos(*f*_0_*t*_0_), *K* is the feedback gain, *D*_0_ is the noise intensity, and *ξ* is the Gaussian noise of the unit standard deviation [Fig 1(A)]. By introducing a new timescale *t* = (λ + *K*)*t*_0_, we can eliminate the prefactor of the term (λ + *K*)*x* which appears on the right hand side, and Eq (1) is transformed to the standard form of the Langevin equation

$$\begin{array}{c} \dot{x}=-x+\epsilon \cos\left(ft\right)+\sqrt{2D}\xi \left(t\right), \end{array}$$ (2)

where *ϵ* = *ϵ*_0_*K*/(λ + *K*), *f* = *f*_0_/(λ + *K*), *D* = *D*_0_/(λ + *K*). The probability density function of the particle position *P*(*x*, *t*) is fully described by the following Fokker-Planck equation:

$$\begin{array}{c} \partial_{t}P\left(x,t\right)=\partial_{x}\left(x-\epsilon \cos\left(ft\right)+D\partial_{x}\right)P\left(x,t\right), \end{array}$$ (3)

where ∂_*x*_ denotes the operator ∂/∂_*x*_. The exact solution of Eq (3) is provided by the Ref. [19] as

$$\begin{array}{c} P\left(x,t\right)=\sqrt{\frac{1}{2\pi D}}\exp\left(\frac{-{\left(x-g\cos(ft+\alpha )\right)}^{2}}{2D}\right), \end{array}$$ (4)

where $g=1/\sqrt{1+f^{2}}$, *α* = arccos(*g*). The analytical form for the reaching success rate *P*_*R*_(*t*), which is the probability that the agent reaches the range of [*x*_*g*_(*t*) − *θ*, *x*_*g*_(*t*) + *θ*] is computed by

$$\begin{array}{c} \left\langle P_{R}\left(t\right)\right\rangle =P_{R}\left(t\right)=\int_{s(t)-\theta }^{s(t)+\theta }P\left(x,t\right)dx=\frac{1}{2}\left(\text{erf}\left(\frac{\theta +B}{\sqrt{2D}}\right)+\text{erf}\left(\frac{\theta -B}{\sqrt{2D}}\right)\right), \end{array}$$ (5)

where *s*(*t*) = *A* cos(*ft*), *A* is the effective amplitude of *x*_*g*_, and is *ϵ*_0_/(λ + *K*), *B* = *ϵc* − *gc*_*α*_, *c* = cos(*ft*), *ϵ* = *ϵ*_0_*K*(λ + *K*), *c*_*α*_ = cos(*ft* + *α*). Note that the analytical form of the ensemble average of *P*_*R*_(*t*) is identical to *P*_*R*_(*t*), that is, 〈*P*_*R*_(*t*)〉 = *P*_*R*_(*t*), where 〈*z*〉 denotes the ensemble average of *z*.

The peak *P*_*R*_(*t*) is calculated using *D* = *R*_*e*_(*θB*/log(±*Q*)), where $Q=\sqrt{\left(-\theta -B)/(\theta -B\right)}$, and *R*_*e*_() denotes taking the real part. This indicates that the optimal noise intensity needed to maximize reaching probability is dependent on the interplay between the geometrical size of the end-effector and the drive frequency.

The probability 〈*P*_*R*_〉 = 〈*P*_*R*_(*t* = 1/*f*)〉 exhibits two distinct modes based on the balance between *θ* and *B*, as shown in Fig 1(F). Surprisingly, lim_*D*→0_〈*P*_*R*_〉 is limited to either 1 or 0, as

$$\begin{array}{c} \lim_{D\to 0}\left\langle P_{R}\right\rangle =\left\{ \begin{array}{cc} 0, & \theta \leq B,\\ 1, & \theta >B \end{array}\right. \end{array}$$ (6)

The condition *θ* ≤ *B* corresponds to an unreachable regime, where the agent cannot reach the target position without the help of noise, and 〈*P*_*R*_〉 is maximized by the optimal noise intensity, as shown in Fig 1(B), 1(C) and 1(F). In contrast, *θ* > *B* corresponds to a reachable regime and 〈*P*_*R*_〉 = 1 by *D* = 0, as shown in Fig 1(D) and 1(F). It may be counterintuitive that 〈*P*_*R*_〉 increases following increases in *f* (i.e., a higher frequency leads to a more reachable condition) (Fig 1(B) and 1(C)). This is because an increase in *f* results in a decrease in *B*, as shown in Fig 1(E), and *θ* ≥ *B* leads to 〈*P*_*R*_〉 > 0.

### Design of simulated neurophysical agent

As an applicative and more general experimental framework for RSRI via GSR, we consider a two-dimensional particle system driven by a nonlinear feedback controller consisting of two arrays of FitzHugh-Nagumo neurons. The motion dynamics of the particle system are described as

$$\begin{array}{c} \dot{v}=-\gamma v+f\left(t\right)+D_{m}\xi_{m}\left(t\right), \end{array}$$ (7)

where ***v*** is the velocity vector of the agent, ***f*** is the force generated by a neural motion controller, *γ* is the friction coefficient and *γ* = 0.6 throughout this article, ***ξ***_*m*_(*t*) is a Gaussian noise vector of unit intensity, and *D*_*m*_ is the motion-noise intensity.

The motion controller is designed to receive a feedback signal ***s***(*t*) and outputs the force ***f***(*t*) based on the neuronal firing rate of two ensembles of FitzHugh-Nagumo (FHN) neurons, one for each dimension, as

$$\begin{array}{c} f\left(t\right)=K(R\left(t\right)-R_{0}), \end{array}$$ (8)

where *K* is the feedback gain, ***R***_0_ is a offset variable, and ***R***(*t*) is the firing rate of the neuron ensemble. The dynamics of the *i*th FHN neuron of the *j*th ensemble is expressed as

$$\begin{array}{ccc} \epsilon \dot{V_{i}^{j}} & = & V_{i}^{j}\left(V_{i}^{j}-1/2\right)\left(1-V_{i}^{j}\right)-W_{i}^{j}+b+s^{j}\left(t\right)+D_{b}\xi_{b,j}, \end{array}$$ (9)

$$\begin{array}{ccc} \dot{W_{i}^{j}} & = & V_{i}^{j}-W_{i}^{j}+\sqrt{2D_{s}}\xi_{i}^{j}\left(t\right), \end{array}$$ (10)

where *ϵ* = 0.005, *b* is the bias signal, *ξ*_*b*_ is a Gaussian noise of unit intensity, *D*_*b*_ is the bias variability and *D*_*b*_ = 0 unless otherwise stated, *s*^*j*^(*t*) is the input feedback signal to the *j*th neuron ensemble, *V* is the fast variable, and *W* is the slow recovery variable. Independently of the motion additive noise *D*_*m*_ ***ξ***_*m*_(*t*), a neuron ensemble receives additive noise *D*_*s*_ ***ξ***(*t*) where ***ξ***(*t*) is the Gaussian noise of the unit standard deviation and *D*_*s*_ is the noise intensity. The firing event $R_{i}^{j}\left(t\right)$ of a neuron is computed by

$$\begin{array}{c} R_{i}^{j}\left(t\right)=\left\{ \begin{array}{cc} 1, & V_{i}^{j}>0.5,\\ 0, & V_{i}^{j}\leq 0.5, \end{array}\right. \end{array}$$ (11)

The mean firing rate of the *j*th ensemble is computed as $R^{j}\left(t\right)=1/N\sum_{i=1}^{N}R_{i}^{j}\left(t\right)$ (*N* = 500 unless otherwise stated). The ensemble is in an excitable regime for *b* ≤ 0.274, and is in an oscillatory regime (i.e., neurons spontaneously fire without any signal input) for 0.274 < *b* < 0.3.

**Fig 2:**
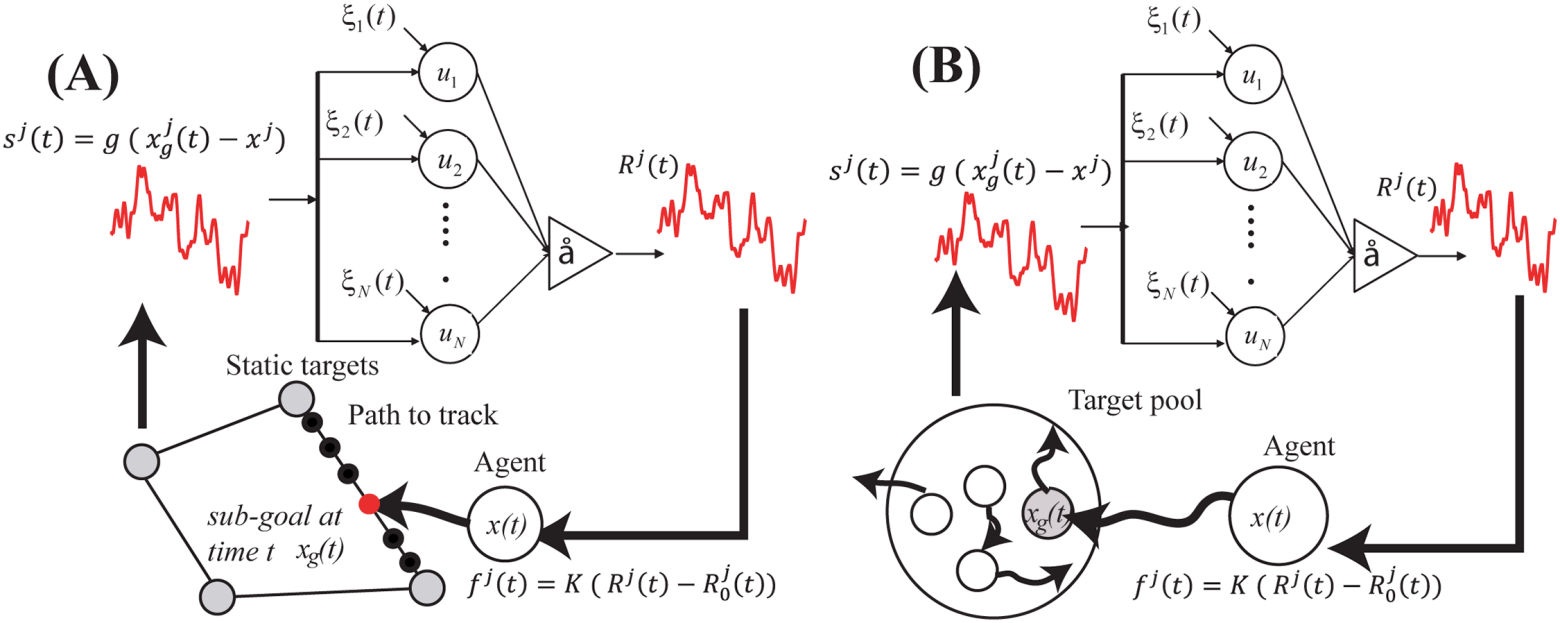
Neurophysical agent design and task setup. **(A, B)** Two different numerical simulation setups for studying behavioral NIO. In setup (A), we study the NIO when a neurophysical agent tracks along a static predesigned path. In setup (B), we study the NIO that occurs when the agent captures randomly moving (i.e., noisy) targets. In the second paradigm, we consider not only the additive neural and force noises internal to the subject agent, but also the motion noise of the moving target.

The input signal ***s***(*t*) encapsulates the computation performed by the neuron ensemble, namely that of a positional PI (proportional-integral) feedback controller, as

$$\begin{array}{ccc} s(t) & = & g(e\left(t\right)+K_{I}\int_{0}^{t}e\left(t^{\prime }\right)dt^{\prime }), \end{array}$$ (12)

$$\begin{array}{ccc} e(t) & = & x_{g}\left(t\right)-x, \end{array}$$ (13)

where *K*_*I*_ is gain for the error-integral control and *g* is the input gain. Furthermore, ***x***(*t*) and ***x***_*g*_(*t*) are the current and desired agent positions, respectively. Note that ***x***_*g*_(*t*) is predesigned in the path-tracking experiment in Fig 2(A), and is dynamically updated at every simulation time-step in the capturing experiment shown in Fig 2(B).

### Numerical simulation of static reaching task

We consider two different kinds of tasks. First, we consider a static goal-reaching task. This task consists of static path planning and path tracking. A static reaching task is the standard motion control task for a biological system, e.g., arm-reaching or eye movement [20–22], and for robot navigation. In this standard goal-reaching task, we assume that the target is fixed. Agent motion fluctuations are influenced by the two noise sources, (1) the motion additive noise *D*_*m*_ ***ξ***_*m*_(*t*), and (2) the neural additive noise *D*_*s*_ ***ξ***(*t*).

In the numerical simulation shown in Fig 2(A), the agent is controlled to visit the four goal positions (*X*_*i*_, *Y*_*i*_) = (cos(1/4*π* + *π*/2*i*), sin(1/4*π* + *π*/2*i*)) (where *i* = 0, 1, …, 3) sequentially, switching to a new goal every *T* [s] ((*i* = 0, 1, …, 3)). When the agent goal is switched to the next one, the line from the agent’s current position ***x***(*t* = *kT*) to the next goal position *t* = (*k* + 1)*T* is equally partitioned into *T*/Δ*t* sub-goals ***x***_*g*_. The agent is controlled to track this pre-designed path ***x***_*g*_(*t*). The offset ***R***_0_ is computed as the time-average of ***R*** of the initial 100s in every task simulation and this initial period is excluded from the performance analysis.

### Performance measures of motion accuracy

Because the agent is scheduled to reach the *k*th goal at *t* = *kT* [s], we compute the distance *d*_*e*_ of the agent position from the *k*th goal every *T* [s], and use *d*_*e*_ as a *linear* measure for the static goal-reaching task. The ensemble average of *d*_*e*_ over different simulation trials, 〈*d*_*e*_〉 is computed as

$$\begin{array}{c} \left\langle d_{e}\right\rangle =\left\langle \frac{1}{M}\sum_{k=1}^{M}{\left({\left(x\left(kT\right)-x_{g}\left(kT\right)\right)}^{2}+{\left(y\left(kT\right)-y_{g}\left(kT\right)\right)}^{2}\right)}^{1/2}\right\rangle . \end{array}$$ (14)

Furthermore, we use the average motion error 〈*e*_*m*_〉 as another *linear* sensorimotor performance index.

$$\begin{array}{c} \left\langle e_{m}\right\rangle =\left\langle {\left(\frac{1}{T_{e}}\int_{0}^{T_{e}}dt{\left(x\left(t\right)-x_{g}\left(t\right)\right)}^{2}+{\left(y\left(t\right)-y_{g}\left(t\right)\right)}^{2}\right)}^{1/2}\right\rangle , \end{array}$$ (15)

where *T*_*e*_ is a sufficiently long time. That is, 〈*e*_*m*_〉 is computed by averaging the error across all simulation time steps.

### A nonlinear performance measure: Goal-reaching success rate

In addition to the measures *d*_*e*_ and *e*_*m*_, we use a measure that is obtained by applying a nonlinear function to *d*_*e*_. A straightforward example of such a nonlinear measure involving using a threshold function to digitize the distance *d*_*e*_ is

$$\begin{array}{c} S_{R}\left(d_{e}\right)=\left\{ \begin{array}{cc} 1, & d_{e}<\theta ,\\ 0, & d_{e}\geq \theta \end{array}\right. \end{array}$$ (16)

where *d*_*e*_ is the distance of the agent from the goal position. Note that the measure *S*_*R*_(*d*_*e*_) is applicable to many biological tasks, such as capturing prey, and to reaching tasks, where a system’s physical body is required to be within a certain range of an object within a certain time period. We use the ensemble average of the goal-reaching success rate 〈*P*_*R*_〉 as a task evaluation measure. $S_{R}^{k}\left(d_{e}\right)$ is calculated every at *t* = *kT* [s], and the ensemble average of *P*_*R*_, 〈*P*_*R*_〉 is computed as

$$\begin{array}{c} \left\langle P_{R}\right\rangle =\left\langle \frac{1}{M}\sum_{k=1}^{M}S_{R}^{k}\left(d_{e}\right)\right\rangle . \end{array}$$ (17)

### Numerical simulation of dynamic capturing task

In the numerical simulation shown in Fig 2(B), we consider a dynamic reaching task where the goal position (i.e., the position of the target objects) moves. In this setup, we use Brownian particles as target objects. Therefore, the motion of the target object provides an additional source of agent motion fluctuation. Note that in the framework (B), there is no pre-designed path to track and the agent goal position is updated at every time step based on the movement of the target object.

In the simulation environment, *N*_*p*_ moving target objects are located randomly within range [−*L*, *L*] (we use *N*_*p*_ = 100 and *L* = 7.5 in this paper), as shown in Fig 2. The *i*th target object moves randomly based on the dynamics

$$\begin{array}{c} m_{g,i}{\dot{v}}_{g,i}=-\gamma v_{g,i}+D_{p}\zeta_{i}\left(t\right)+F_{w}, \end{array}$$ (18)

where *m*_*g*_ = 0.1, ***v***_*g*,*i*_ is the velocity, ***ζ***_*i*_(*t*) is the Gaussian noise of unit variance, and *D*_*p*_ is the noise intensity. The target object is subjected to a force *F*_*w*_ from a virtual wall when it moves beyond the region [−*L*, *L*].

The agent is controlled to pursue the position of a moving target. That is, ***x***_*g*_(*t*) = ***x***_*p*_(*t*)+***v***_*p*_(*t*)Δ*t*, where ***x***_*p*_(*t*) and ***v***_*p*_(*t*) are the position and the velocity of the current moving target, respectively. When the distance *d*_*p*_ between the agent and the target satisfies *d*_*p*_ < *θ*, the moving target is “captured” and is removed from the simulation. After the agent captures a certain target, the target is switched to the nearest moving object. The offset ***R***_0_ is computed as the time-average of ***R*** of the initial 300s in every task simulation and this initial period is excluded from the performance analysis.

The capturing rate per unit time is *C*_*r*_, which provides a nonlinear measure of the agent’s sensorimotor performance on this task. This is computed as

$$\begin{array}{c} C_{r}=\frac{1}{T}\int_{0}^{T}\Theta \left(\theta -d_{p}\right)dt, \end{array}$$ (19)

where Θ(*z*) is the Heaviside step function.

## Results

### Benefits of noise in the behavior of a neurophysical agent

**Fig 3:**
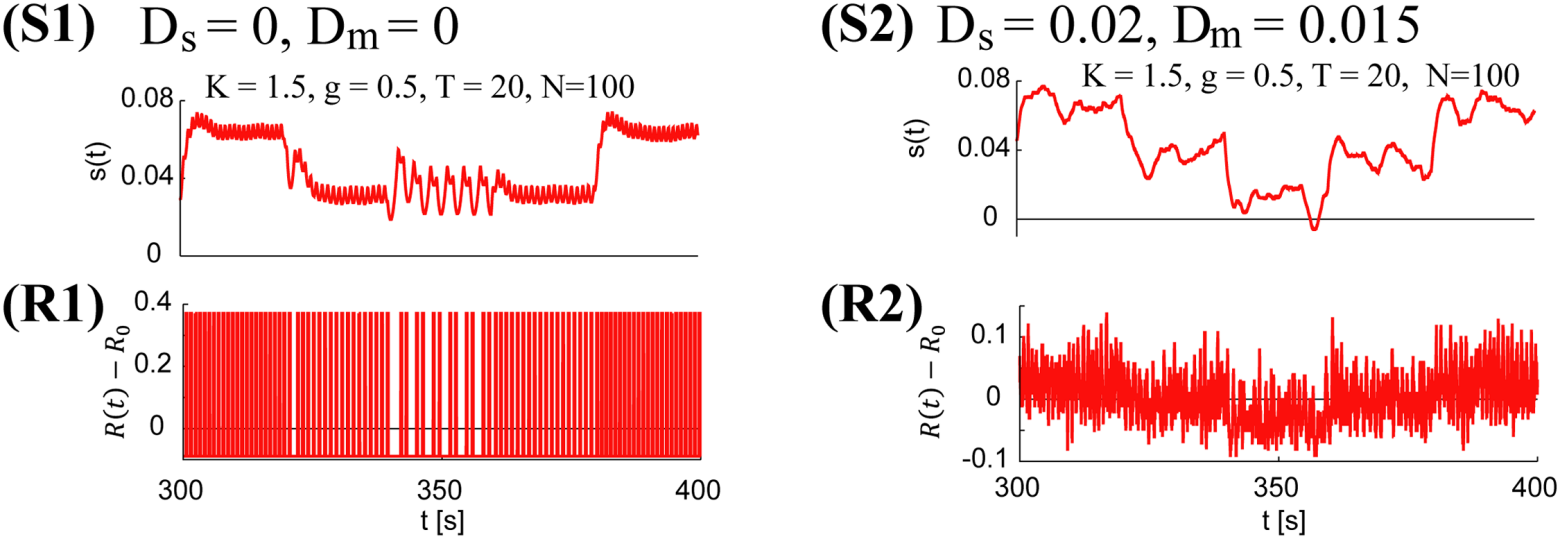
Emergent aperiodic control signal and asynchronous neural firing. **(S1, R1)** The input signal to the motion actuator **(S1)** and the corresponding neural firing rate *R*(*t*) − *R*_0_ **(R1)**, with *D*_*s*_ = 0 and *D*_*m*_ = 0. Note that the input signal to the actuator is totally deterministic, although it exhibits jittering. In addition, the corresponding neural spikes are synchronized (the even vertical lines represent bursts of spikes, not individual spikes.) **(S2, R2)** An aperiodic and stochastic control signal emerges with either *D*_*m*_ > 0 or *D*_*s*_ > 0 **(S2)**. The corresponding firing rate becomes asynchronous if *D*_*s*_ > 0 **(R2)**.

For *D*_*s*_ = 0 and *D*_*m*_ = 0, the system exhibits fully deterministic behavior, although it may exhibit jittering due to the overshooting characteristics of a simple feedback controller. In this deterministic regime, the neural system exhibits a totally synchronized firing pattern across different initial neuronal conditions (see Fig 3 for both the motion-control signal [S1, S2] and the neural firing-rate time series [R1, R2]). Clearly, in this regime, neural spike frequency encodes the motion control signal. The agent-movement and motion-control signals become stochastic and aperiodic with either *D*_*m*_ > 0 or *D*_*s*_ > 0. For relatively large *D*_*s*_ values, the neuronal firing rate also becomes asynchronous. Note that the combination *D*_*m*_ > 0 and *D*_*s*_ = 0 can generate an aperiodic motion-control signal, but it cannot generate an asynchronous neuronal firing rate.

#### Motion accuracy improvement by SR in a neural motion controller

**Fig 4:**
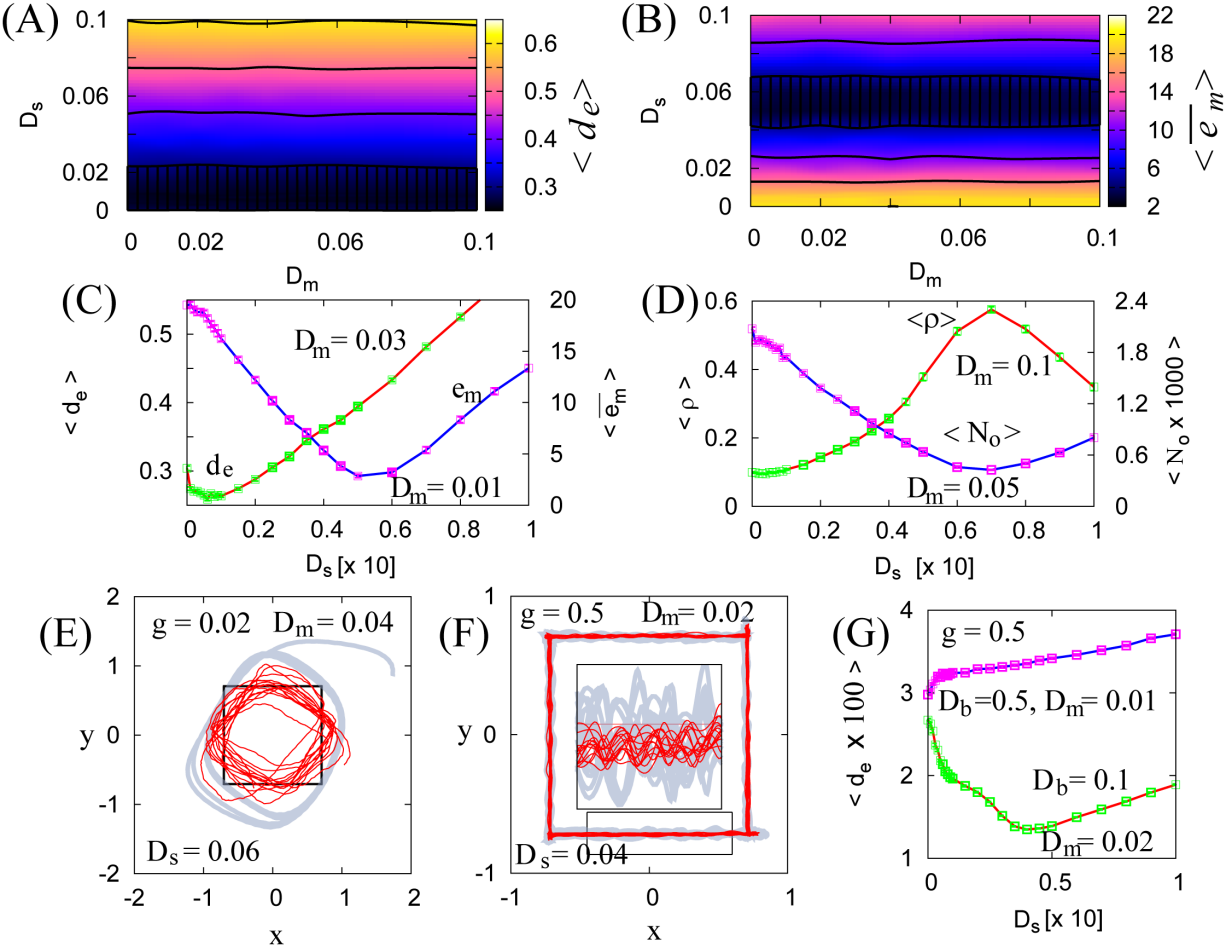
Motion change due to the presence of neural and force noises. **(A, B)** 〈*d*_*e*_〉 and $\langle \bar{e_{m}}\rangle$ with the parameters *T* = 10, *K* = 10, *g* = 0.02. **(C)** 〈*d*_*e*_〉 with *D*_*m*_ = 0.01 and 〈*e*_*m*_〉, with *D*_*m*_ = 0.05 as a function of *D*_*s*_. **(D)** 〈*ρ*〉 and 〈*N*_*o*_〉 as a function of *D*_*s*_. **(E, F)** The change in motion trajectory due to the presence of neural and motion noises, with *T* = 10, *K* = 10, and *g* ≪ 1 [**(E)**] and *g* ≫ 0 [**(F)**]. The inset is an enlargement of the respective areas inside the rectangles. Note that the bias variability *D*_*b*_ ≫ 0 leads to high pooling ability and reduces the oscillatory motion, but obscures the neuronal SR effect. Furthermore, the motion accuracy achieved due to neuronal SR (with *D*_*b*_ ∼ 0 and *D*_*s*_ > 0) is higher than it is in the noiseless system with high motor pooling ability (with *D*_*b*_ ≫ 0 and *D*_*s*_ = 0) **(G)**. Numerical 〈*d*_*e*_〉, $\langle \bar{e_{m}}\rangle$, 〈*ρ*〉, and 〈*N*_*o*_〉 are computed from 500 trials of a 500 s numerical simulation.

Fig 4 shows that the best motion accuracy, i.e., the minimum 〈*d*_*e*_〉 or the minimum 〈*e*_*m*_〉, is realized when there is nonzero neural noise *D*_*s*_ [Fig 4(A)–4(C)]. Interestingly, the peak positions for 〈*d*_*e*_〉 and 〈*e*_*m*_〉 are very different (i.e., the minimum 〈*d*_*e*_〉 is calculated using *D*_*s*_ ≈ 0.01, and the minimum 〈*e*_*m*_〉 is calculated using *D*_*s*_ ≈ 0.06).

The measure 〈*e*_*m*_〉 has a strong dependency on the neural performance measures *ρ*, the correlation coefficient of the input control signal and the neuronal firing rate, and *N*_*o*_, the time-averaged product of the ***s***(*t*) norm and ***R***(*t*) norm. Here, *ρ* and *N*_*o*_ are computed as $\rho =1/T_{e}\int_{0}^{T_{e}}\left(s\left(t\right)\cdot R\left(t\right)\right)dt/N_{o}$ and $N_{o}=1/T_{e}\int_{0}^{T_{e}}\left(∥s\left(t\right)∥∥R\left(t\right)∥\right)$ dt.

**Fig 5:**
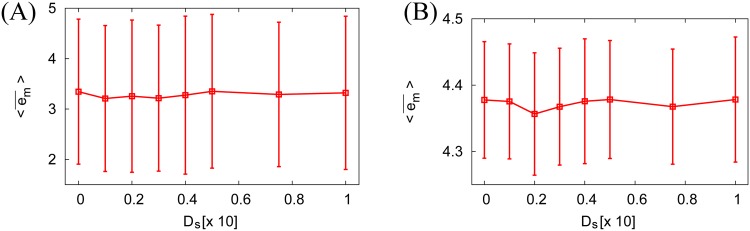
Motion error with respect to *D*_*s*_. $\langle \bar{e_{m}}\rangle$ with *g* = 0.02, *D*_*b*_ = 0.1, and *D*_*s*_ = 0.05 for **(A)**, and *g* = 0.5, *D*_*b*_ = 0.1, and *D*_*s*_ = 0.005 for **(B)**. The error bars indicate standard deviations. Note that we could not find any significant improvements due to the presence of force noise *D*_*m*_. Numerical $\langle \bar{e_{m}}\rangle$ is computed from 500 trials of a 500 s numerical simulation, and the error bars correspond to the standard error.

We can see that there exist two kinds of SR-based motion accuracy improvements: a subthreshold SR corresponding to a small input gain *g* [Fig 4(E)], and a suprathreshold SR corresponding to a large input gain *g* [Fig 4(F)]. With the small input gain *g* ∼ 0, the agent motion tends to overshoot the desired path due to the poor information transmission to the motion controller [Fig 4(E), bold gray line for *D*_*s*_ = 0 and solid red line with *D*_*s*_ = 0.06]. In contrast, with the large input gain *g* ≫ 0, the agent motion tends to oscillate around the desired path due to the hard synchronization of neuronal firing [Fig 4(F), bold gray line for *D*_*s*_ = 0]. This synchronized neuronal firing is due to the poor pooling ability of the motor controlling neurons (the panel **(F)** is obtained using *D*_*b*_ = 0.1). The large bias variability *D*_*b*_ ≫ 0 can improve the pooling ability in the noiseless neural controller *D*_*s*_ = 0. However, it must be noted that *D*_*b*_ ≫ 0 obscures the SR effect for *D*_*s*_ > 0. Furthermore, the motion accuracy provided by SR is higher than the motion accuracy realized using noiseless pooling with *D*_*b*_ ≫ 0 and *D*_*s*_ = 0, as shown in panel **(G)**. Note that *g* ≫ 0 is exactly the case wherein SR growth [18] occurs. Furthermore, we could not find any motion accuracy improvements due to the presence of force noise (Fig 5).

### Improvement in static reaching success rate

**Fig 6:**
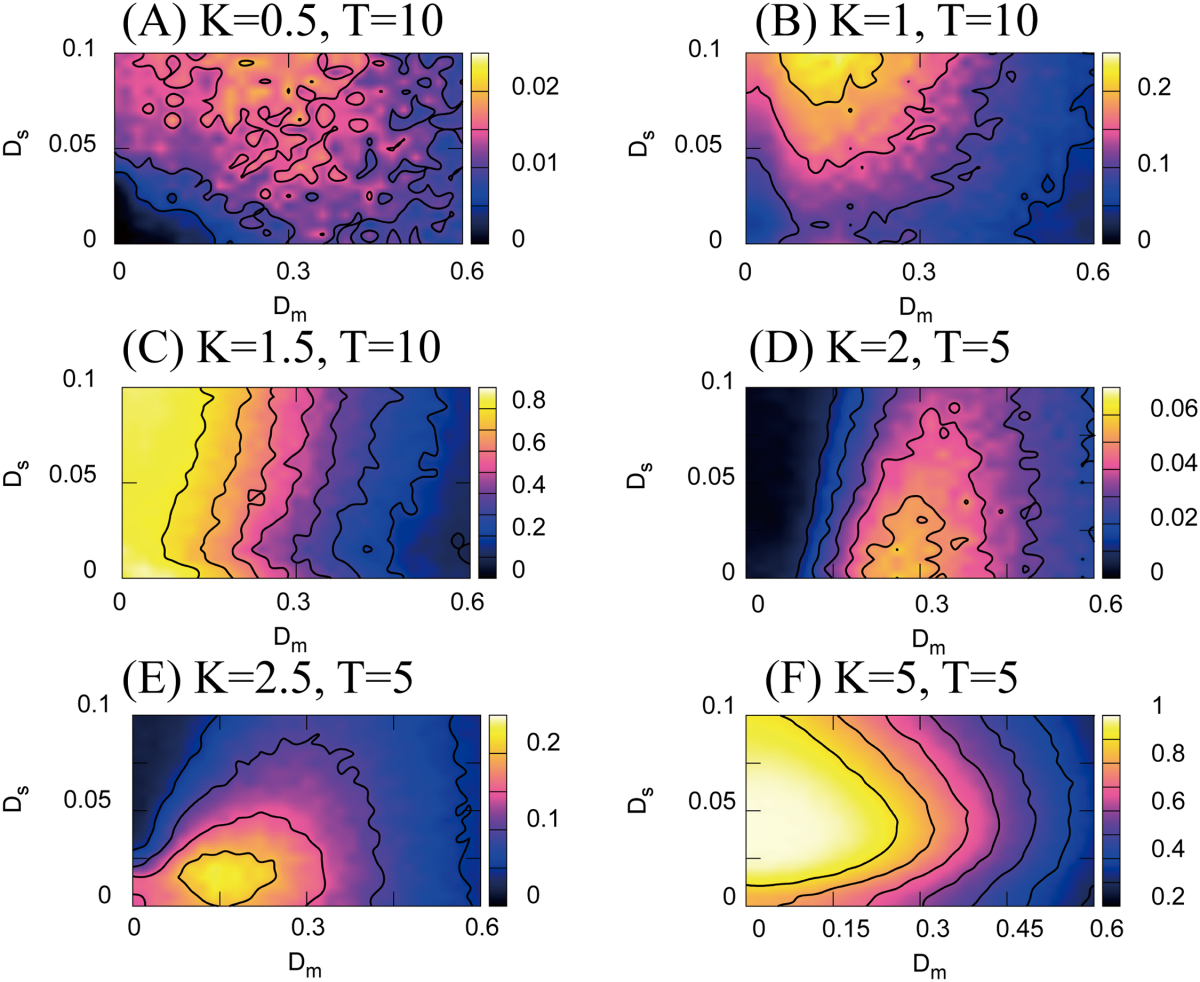
The goal-reaching success rate 〈*P*_*R*_〉 as a function of motion noise *D*_*m*_ and neural noise *D*_*s*_. The task parameters are *T* = 10, *K* = 0.5 − 1.5 in panels **(A–C)**, and *T* = 5, *K* = 2 − 5 in panels **(D–F)**. Additive motion noise improves reaching success rate when *K* is not sufficient to produce a 100% goal-reaching success rate (this is shown in panels **(A), (B), (D), and (E)**). As shown in panels **(C)** and **(F)**, if *K* is large enough to realize a 100% success rate, the *P*_*R*_ monotonically decreases with *D*_*m*_. Numerical 〈*P*_*R*_〉 are computed from 100 trials of a 500 s numerical simulation with *θ* = 0.1 and *N* = 100, and the error bars correspond to the standard error.

The distance *d*_*e*_, which was used in the previous study, represents the “linear” difference between the agent and the position of the goal. Because this difference is linear, *d*_*e*_ exhibits a monotonic increase in response to the intensity of the additive force noises. This implies that if we use a certain measure with a nonlinear dependence on the agent and goal positions, we may observe benefits of motion noise in the sensorimotor task. In fact, as expected from the theory of GSR, we observe the benefits of the noise when we use reaching success rate as a measure, as shown in Fig 6.

Fig 6 shows the goal-reaching success rate as a function of the neural and motion additive noises *D*_*s*_ and *D*_*m*_ in the experimental setup shown in Fig 2(A). With the parameter sets shown in Fig 6(A), 6(B), 6(D) and 6(E), the agent cannot achieve a good reaching success rate using the default deterministic feedback control because force feedback gain *K* is not sufficient. In this “deterministically unreachable” parameter region, the combination of nonzero neural noise and nonzero motion noise leads to an improvement in the goal-reaching success rate. This realization of RSRI can be interpreted as a result of the interplay between the motion noise and the neural noise: motion noise generates an aperiodic input signal in the neural system, as shown in Fig 3(S1), 3(R1), 3(S2) and 3(R2), and neural noise generates an aperiodic neural SR [16–18]. Note: in the deterministically reachable region in Fig 6(C) and 6(F) (i.e., *P*_*R*_ > 0 for *D*_*s*_ = 0 and *D*_*m*_ = 0), the *P*_*R*_ improvement effect due to motion noise is in principle unobservable (because *P*_*R*_ = 1 by default).

**Fig 7:**
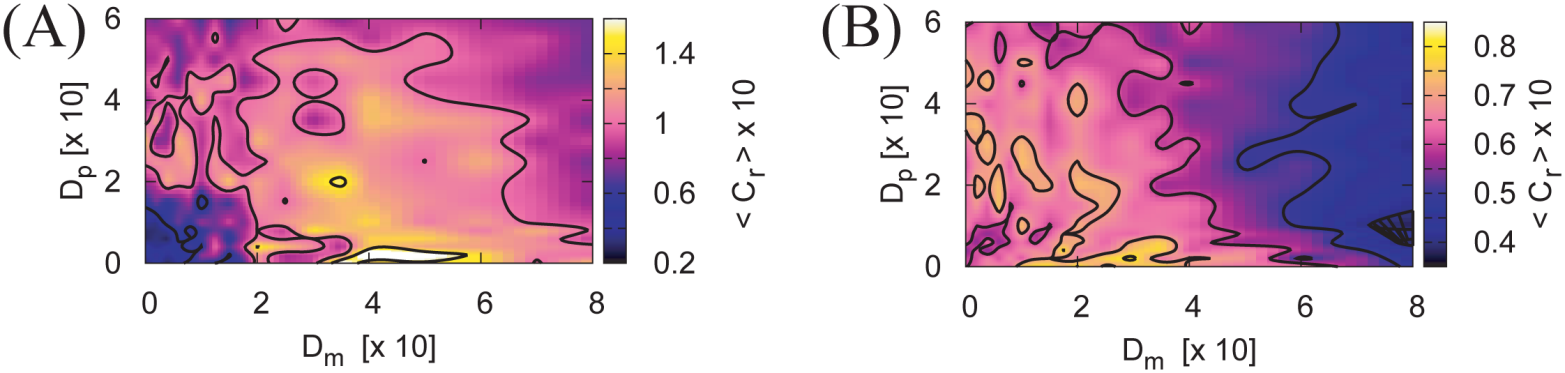
Improvement in capture rate 〈*C*_*r*_〉 due to motion noise *D*_*m*_ and the noise of the targets motion *D*_*p*_. The ensemble average of the capture rate 〈*C*_*r*_〉 as a function of *D*_*p*_ and *D*_*m*_ with internal neural noise *D*_*s*_ = 1 × 10^−3^ **(A)** and *D*_*s*_ = 5 × 10^−3^ **(B)**. The other parameters are *K* = 5, *g* = 10^−2^, *b* = 0.24, *N* = 100, and *θ* = 1. The peak 〈*C*_*r*_〉 is distributed roughly along the line *D*_*p*_ + *D*_*m*_ = 0.4 in (A) and *D*_*p*_ + *D*_*m*_ = 0.3 in (B). It is clear that the maximization of 〈*C*_*r*_〉 requires a balance among *D*_*m*_, *D*_*s*_, and *D*_*p*_. Numerical 〈*C*_*r*_〉 are computed from 400 trials of a numerical simulation.

### Improvement in the capturing rate due to environment-agent noise balancing

In the dynamic reaching experiment, we consider a task where the agent is controlled to capture moving prey. Furthermore, we investigate how the capture number per unit of time depends on the motion fluctuations of the agent and those of the moving targets.

The dependence of capture rate 〈*C*_*r*_〉 on *D*_*m*_ and *D*_*p*_ is shown in Fig 7(A) and 7(B), with *K* = 0.5 and *θ* = 1. Clearly, 〈*C*_*r*_〉 is improved with the presence of motion additive noise *D*_*m*_. Interestingly, 〈*C*_*r*_〉 is also improved with the presence of *D*_*p*_, which indicates the prey’s motion noise. Furthermore, 〈*C*_*r*_〉 is a function of *D*_*p*_, *D*_*m*_, and *D*_*s*_. These interesting results imply that the ability to capture is a function not only of agent motion and neural noise, but also of the prey’s motion noise. From the point of view of the capturing agent, the neural and motion noises must be adjusted to match the intensity of the prey’s motion noise. On the other hand, for the prey to avoid being captured, it must adjust the intensity of its motion noise away from that of the agents. This experimental result implies that biological systems in the context of survival competition will control their neural and motion noise intensities based on their environmental noise levels.

**Fig 8:**
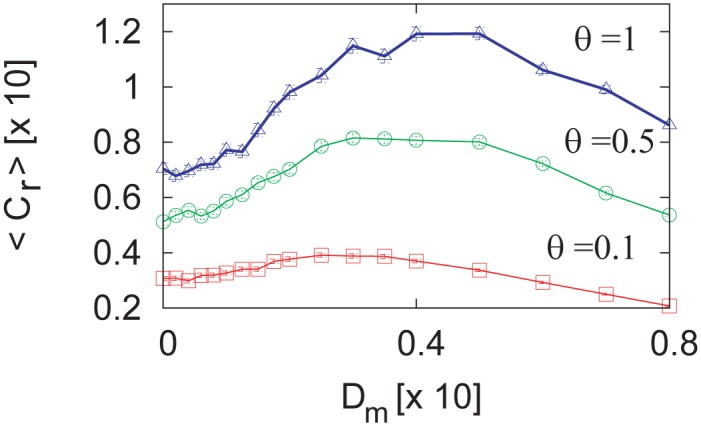
Capture rate modification by threshold size *θ*. The parameters are *D*_*s*_ = 1 × 10^−3^, *K* = 5, *b* = 0.24, *N* = 100, and *g* = 0.01. The rate of improvement in capture rate is dependent on the size of the geometric threshold *θ*. Numerical 〈*C*_*r*_〉 are computed from 100 trials of a 500 s numerical simulation, and error bars indicate standard errors and are within the symbols.

The improvement in the capturing rate 〈*C*_*r*_〉 due to *D*_*m*_ is dependent on the size of the geometric threshold *θ*, as shown in Fig 8. It may be reasonable to presume that a larger biological agent can more efficiently exploit the GSR that is induced by motion fluctuations when capturing small targets.

Ref. [4–6] report that the feeding rate of paddlefish (capturing rate of planktons per minute) is improved in the presence of electrical sensory noise. Conventionally, this feeding behavior improvement has been thought to be the result of sensitivity improvement due to the presence of electrical sensory noise. Our results may imply that, in addition, the electrical signal noise induces motion fluctuations in the capturing agent, which then help to improve the capture rate.

The effect of motion noise on the capturing task is summarized as follows: motion noise enables reaching that is not obtainable deterministically, and the optimal motion noise intensity is determined by a balance with the target motion noise.

### Dynamic capturing rate improvement using a simple PI feedback controller

To determine whether our results have general applicability, we obtained experimental data using a simple PI motion controller that did not have any neurons. The force output of this simple PI motion controller is described as

$$\begin{array}{c} f\left(t\right)=K(e\left(t\right)+K_{I}\int_{0}^{t}e\left(t^{\prime }\right)dt^{\prime }), \end{array}$$ (20)

where ***e***(*t*) = ***x***_*g*_(*t*) − ***x***.

**Fig 9:**
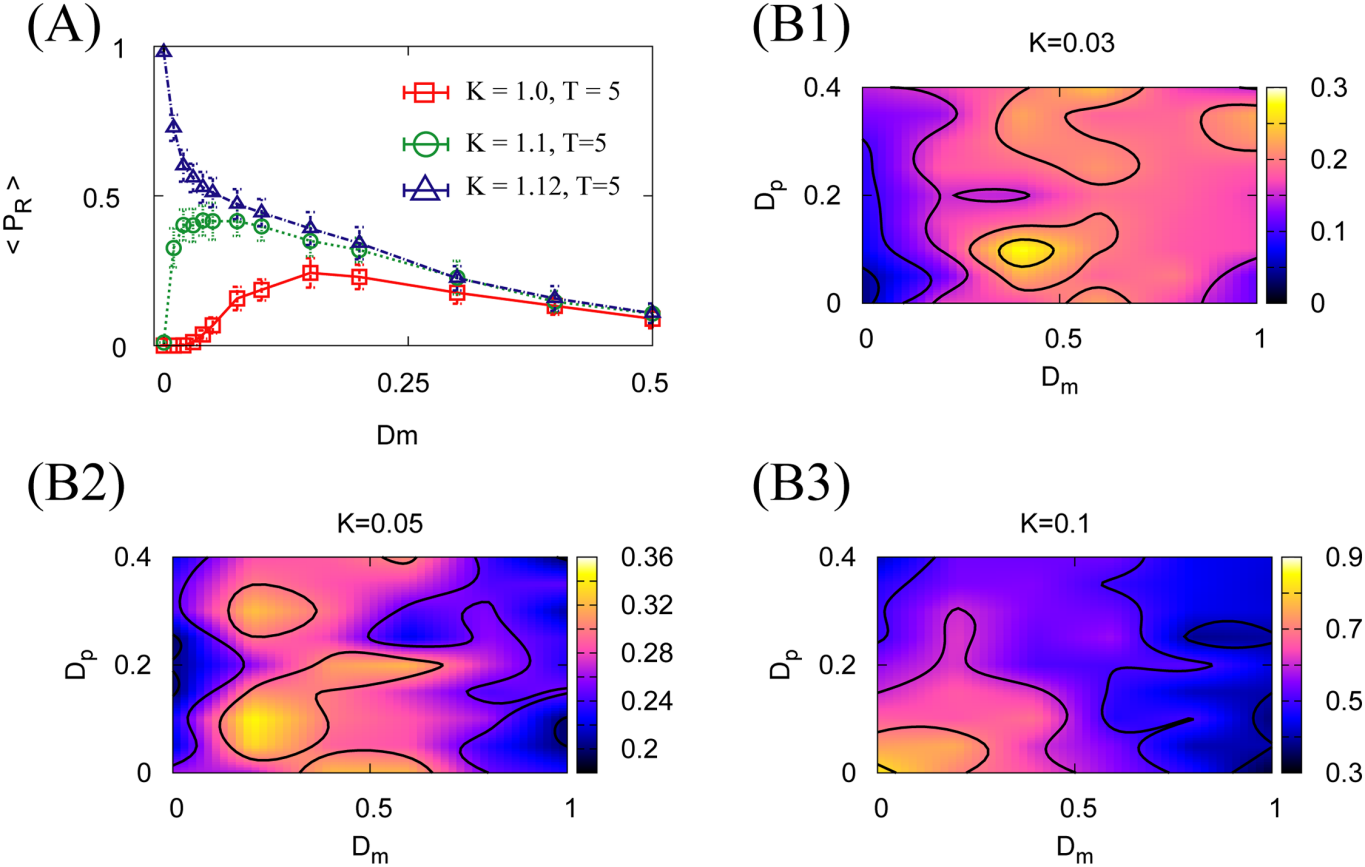
Behavioral SR of an agent driven by a simple non-neural PI controller. **(A)** Improvement in the goal-reaching success rate due to additive motion noise. Numerical 〈*P*_*R*_〉 are computed from 40 trials of a 500 s numerical simulation with *K*_*I*_ = 0.01 and *θ* = 0.1. Error bars in **(A)** indicate standard deviations. **(B1–B3)** Capture-rate improvement due to motion additive noise. Numerical 〈*C*_*r*_〉 are computed from 1,000 trials. The parameters for **(B1–B3)** are *K*_*I*_ = 0.02 × 10^−2^ and *θ* = 2.

Although several parameter adjustments are required, it is possible to reproduce the results of Figs 7 and 8. Fig 9(A) shows the improvement in reaching success rate, and Fig 9(B1)–9(B3) show the improvement in the capture rate. These results support the idea that behavioral SR in reaching and capturing tasks is a general property of feedback-controlled physical agents.

## Discussion

We investigated NIO in the context of sensorimotor coordination in a neurophysical 2D particle agent. The motion controller of the agent consisted of an FHN neuron ensemble. The addition of neural noise to the controller led to an improvement in the agent’s motion accuracy, as shown in Fig 4(A)–4(C). The motion accuracy improvement by the addition of neural noise would be primarily a consequence of the neural SR that optimizes the controller feedback output, i.e., the maximization of *ρ* as shown in Fig 4(D). It must be noted that the addition of neural noise decreases the variance of a neural output [i.e., 〈*N*_*o*_〉 shown in Fig 4(D) decreases by the addition of neural noise]. Because the addition of force noise to the agent body monotonically degraded motion accuracy, as shown in Fig 5, motion accuracy improvement by the addition of neural noise would be a secondary consequence of the decrease in the neural output variance.

Although motion fluctuations per se degrade motion accuracy, we found that a *nonlinear* performance measure such as goal-reaching success rate can exhibit the emergence of an NIO induced by the the motion fluctuations. Particularly, we found that motion fluctuations improved the goal-reaching success rate 〈*P*_*R*_〉, as shown in Figs 6–9.

Interestingly, for a neurophysical agent 〈*P*_*R*_〉 was a function of force additive noise intensity *D*_*m*_ and neural additive noise *D*_*s*_ as shown in Fig 6. Furthermore, in a capturing task where not only the neurophysical agent but also the prey’s motion was noisy, the capturing success rate 〈*C*_*r*_〉 was a function of agent force noise intensity *D*_*m*_, neural noise intensity *D*_*s*_, and prey’s motion noise intensity *D*_*p*_ as shown in Figs 7 and 9. These results imply that biological systems may handle the balancing of motion and neural noise dependently on the environmental noise.

### Task success ratio as a marker of NIO

It should be noted that we did not find any benefit of motion fluctuation based on measures of the relationship between predesigned paths and the actual motion trajectories. Likewise, we could not find SR-like benefits using measures of the distances among the goal positions and the final agent positions. Only when using measures of the discretized state probability, such as goal-reaching success rate and capturing rate, did we observe SR phenomena induced by motion fluctuation.

It is worth noting that most of the conventional systems for studying SR require a discretized dynamical representation or a digitized output. These systems include traditional double-well potential systems, threshold systems, the spiking neuron, and two-state dynamical systems (e.g., the FitzHugh-Nagumo neuron). These systems are capable of digitizing the input signals or generating digitized output (for reviews of the conventional SR-capable systems and frameworks, see Refs. [11, 23–26]).

Furthermore, the conventional framework for detecting a weak signal requires a discretized measure (i.e., detection or no-detection). In behavioral frameworks, responding to weak and subtle sensory signals also requires a digitized response (i.e., response or no-response). A recent concept for studying SR, called entropic SR [27] and GSR [28, 29], posits that Brownian particles move between two rooms connected by a narrow aperture. In this case, the setup of the two rooms provides the state digitization. (Note that the mechanism of GSR described in Eq (5) would be very different from the conventional mechanism of GSR reported in Refs. [28, 29], although both studies share the common characteristic that the interaction of noise and geometric constraints induce NIO). In this manner, a digitized measure, or digitizing dynamics, may be implicit requisites for observation of the SR. At present, this idea is only an inference from analogy and requires theoretical analysis.

Measures of discretized-state probabilities, such as the goal-reaching success rate and capturing rate can be generalized as task success ratios. It should be noted that the measure “task success ratio” is a highly nonlinear function of a variety of arguments, such as the appropriateness of feedback gain, neural noise intensity, neuron size, input signal gain, input-output information, and the distance from the goal position. Therefore, the task success rate may be able to implicitly represent the extent to which calculations of these arguments are improved by some intervention.

### Conclusion

In this paper, we investigated the prospective benefits of the bodily motion fluctuations of an embodied physical agent. We considered a static path-tracking task and a dynamic capturing task for moving prey agents. We found that motion fluctuations degrade motion accuracy, but improve the reaching success rate and capturing rate. These results imply that a biological agent may exploit bodily motion fluctuation in several behavioral tasks, such as reaching, capturing, and navigation, by adjusting the intensity of the motion noise.

## References

[1] Söderlund G, Sikström S, Smart A. Listen to the noise: Noise is beneficial for cognitive performance in ADHD. Journal of Child Psychology and Psychiatry. 2007;48(8):840–847. doi: 10.1111/j.1469-7610.2007.01749.x 17683456 10.1111/j.1469-7610.2007.01749.x

[2] Usher M, Feingold M. Stochastic resonance in the speed of memory retrieval. Biological Cybernetics. 2000;83:L11–L16. doi: 10.1007/PL00007974 11130587 10.1007/PL00007974

[3] Kitajo K, Nozaki D, Ward LM, Yamamoto Y. Behavioral stochastic resonance within the human brain. Physical Review Letters. 2003;90(21):218103 doi: 10.1103/PhysRevLett.90.218103 12786595 10.1103/PhysRevLett.90.218103

[4] Russell DF, Wilkens LA, Moss F. Use of behavioral stochastic resonance by paddle fish for feeding. Nature. 1999;402(6559):291–294. 10580499 10.1038/46279

[5] Freund JA, Kienert J, Schimansky-Geier L, Beisner B, Neiman A, Russell DF, et al Behavioral stochastic resonance: How a noisy army betrays its outpost. Physical Review E. 2001;63:031910 doi: 10.1103/PhysRevE.63.031910 10.1103/PhysRevE.63.031910 11308681

[6] Freund JA, Schimansky-Geier L, Beisner B, Neiman A, Russell DF, Yakusheva T, et al Behavioral stochastic resonance: How the noise from a *Daphnia* swarm enhances individual prey capture by Juvenil paddlefish. Journal of Theoretical Biology. 2002;214(1):71–83. Erratum in: J. Comp. Neurosci. 2014;37(3):593–594. doi: 10.1006/jtbi.2001.2445 11786033 10.1006/jtbi.2001.2445

[7] Rolls ET. Emotion and Decision Making Explained. 1st ed Oxford; New York, NY: Oxford University Press; 2014.

[8] Brunel N, Wang XJ. Effects of neuromodulation in a cortical network model of object working memory dominated by recurrent inhibition. Journal of Computational Neuroscience. 2001;11(1):63–85. doi: 10.1023/A:1011204814320 11524578 10.1023/a:1011204814320

[9] Hasselmo ME, McGaughy J. High acetylcholine levels set circuit dynamics for attention and encoding and low acetylcholine levels set dynamics for consolidation. Progress in Brain Research. 2004;145:207–231. doi: 10.1016/S0079-6123(03)45015-2 14650918 10.1016/S0079-6123(03)45015-2

[10] Wiesenfeld K, Moss F. Stochastic resonance and the benefits of noise: from ice ages to crayfish and SQUID. Nature. 1995;373(5):33–36. doi: 10.1038/373033a0 7800036 10.1038/373033a0

[11] Moss F, Ward LM, Sannita WG. Stochastic resonance and sensory information processing: a tutorial and review of application. Clinical Neurophysiology. 2004;115(2):267–281. doi: 10.1016/j.clinph.2003.09.014 14744566 10.1016/j.clinph.2003.09.014

[12] Ward LM, Neiman A, Moss F. Stochastic resonance in psyhophysics and in animal behavior. Biological Cybernetics. 2002;87(2):91–101. doi: 10.1007/s00422-002-0328-z 12181585 10.1007/s00422-002-0328-z

[13] Henning MH, Kerscher NJ, Funke K, Wörgötter F. Stochastic resonance in visual cortical neurons: Does the eye-tremor actually improve visual acuity? Neurocomputing. 2002;44–46:115–120. doi: 10.1016/S0925-2312(02)00371-5

[14] Gammaitoni L, Menichella-Saetta E, Santucci S, Marchesoni F, Presilla C. Periodically time-modulated bistable systems: Stochastic resonance. Physical Review A. 1989;40:2114 doi: 10.1103/PhysRevA.40.2114 10.1103/physreva.40.2114 9902370

[15] Hänggi P, Jung P, Zerbe C, Moss F. Can colored noise improve stochastic resonance? Journal of Statistical Physics. 1993;70(1-2):25–47. doi: 10.1007/BF01053952

[16] Collins JJ, Chow CC, Imhoff TT. Aperiodic stochastic resonance in excitable systems. Physical Review E. 1995;52(4):R3321 doi: 10.1103/PhysRevE.52.R3321 10.1103/physreve.52.r3321 9963950

[17] Collins JJ, Chow CC, Imhoff TT. Stochastic resonance without tuning. Nature. 1995;376(6337):236–238. doi: 10.1038/376236a0 7617033 10.1038/376236a0

[18] Yonekura S, Kuniyoshi Y, Kawaguchi Y. Growth of stochastic resonance in neuronal ensembles with the input signal intensity. Physical Review E. 2012;86:011922 Erratum in: Phys Rev E. 2016;93(3–2):039903. doi: 10.1103/PhysRevE.86.011922 10.1103/PhysRevE.86.011922 23005467

[19] Gang H, Nicolis G, Nicolis C. Periodically forced Fokker-Planck equation and stochastic resonance. Physical Review A. 1990;42(4):2030–2041. doi: 10.1103/PhysRevA.42.2030 10.1103/physreva.42.2030 9904251

[20] Faisal AA, Selen LPJ, Wolpert DM. Noise in the nervous system. Nature Reviews Neuroscience. 2008;9(4):292–303. doi: 10.1038/nrn2258 18319728 10.1038/nrn2258 PMC2631351

[21] Harris CM, Wolpert DM. Signal-dependent noise determines motor planning. Nature. 1998;394(6695):780–784. doi: 10.1038/29528 9723616 10.1038/29528

[22] Franklin DW, Wolpert DM. Computational mechanisms of sensorimotor control. Neuron. 2011;72(3):425–442. doi: 10.1016/j.neuron.2011.10.006 22078503 10.1016/j.neuron.2011.10.006

[23] Bulsara AR, Gammaitoni L. Tuning in to noise. Physics Today. 1996;49(3):39–45. doi: 10.1063/1.881491

[24] Gammaitoni L, Hänggi P, Jung P, Marchesoni F. Stocahstic Resonance. Reviews of Modern Physics. 1998;70(1):223–287. doi: 10.1103/RevModPhys.70.223

[25] McDonnel MD, Abbott D. What is stochastic resonance? Definitions, misconceptions, debates, and its relevance to biology. PLOS Computational Biology. 2005;5(5):e1000348 doi: 10.1371/journal.pcbi.1000348 10.1371/journal.pcbi.1000348 PMC2660436 19562010

[26] Lindner B. Coherence and Stochastic Resonance in Nonlinear Dynamical Systems. Humboldt-Universität zu Berlin; 2002.

[27] Burada PS, Schmid G, Reguera D, Vainstein MH, Rubi JM, Hänggi P. Entropic Stochastic Resonance. Physical Review Letters. 2008;101:130602 doi: 10.1103/PhysRevLett.101.130602 18851431 10.1103/PhysRevLett.101.130602

[28] Ghosh PK, Marchesoni F, Savel’ev SE, Nori F. Geometric Stochastic Resonance. Physical Review Letters. 2010;104:020601 doi: 10.1103/PhysRevLett.104.020601 20366581 10.1103/PhysRevLett.104.020601

[29] Zheng C, Guo W, Du L, Mei D. A new model of geometry-induced stochastic resonance. Europhysics Letters. 2014;105(6):60004 doi: 10.1209/0295-5075/105/60004

